# Role of cardiac magnetic resonance in MINOCA of unclear etiology: a case report of a suspicious paradoxical coronary embolism

**DOI:** 10.1259/bjrcr.20220114

**Published:** 2023-02-07

**Authors:** Francesca Scabbia, Michela Zerbini, Lucia Pirani, Riccardo Righi, Monica Viola, Ada Collevecchio, Roberto Rizzati, Biagio Sassone

**Affiliations:** 1 Department of Morphology, Section of Diagnostic Imaging, Surgery and Experimental Medicine, University of Ferrara, via Savonarola 9, 44121, Ferrara, Italy; 2 Department of Radiology, Azienda Unità Sanitaria Locale di Ferrara, via Arturo Cassoli 30, 44121, Ferrara, Italy; 3 Department of Emergency, Division of Cardiology, Delta Hospital, Azienda Unità Sanitaria Locale di Ferrara, via Valle Oppio 2, 44023, Lagosanto, Ferrara, Italy; 4 Department of Emergency, Division of Cardiology, SS.ma Annunziata Hospital, Azienda Unità Sanitaria Locale di Ferrara, via Giovanni Vicini 2, 44042, Cento, Ferrara, Italy; 5 Department of Translational Medicine, University of Ferrara, via Savonarola 9, 44121, Ferrara, Italy

## Abstract

The acronym MINOCA (Myocardial Infarction with Non-Obstructive Coronary Arteries) refers to myocardial infarction with normal or near-normal coronary arteries on invasive angiography. The broad spectrum of pathological mechanisms responsible for myocardial injury in MINOCA makes defining the exact underlying etiology challenging. We report the uncommon case of an acute myocardial infarction with normal coronary arteries suggestive of MINOCA caused by paradoxical coronary embolism due to a wide right-to-left shunting through a patent fossa ovalis. Integrated multimodality imaging diagnostic work-up, including cardiac magnetic resonance, transesophageal contrast echocardiography, and transcranial contrast Doppler, has been crucial for identifying the most likely mechanism underlying MINOCA.

## Case presentation

A 68-year-old female was admitted to the Coronary Care Unit after being evaluated at the Emergency Room for sudden-onset oppressive chest pain lasting 15 min and radiating to the back combined with subtle nonspecific ST-segment changes at the lateral site on a 12-lead electrocardiogram (ECG) and elevated high-sensitive troponin I (peak value 3700 ng l^−1^, normal value for female<12 ng l^−1^). She had active smoking and hypertension as cardiovascular risk factors. On admission, the patient was asymptomatic, physical examination was unremarkable, and vital parameters were within normal limits; laboratory tests showed normal values for both blood cell counts and C-reactive protein; a nasopharyngeal swab was obtained resulting negative for severe acute respiratory syndrome-coronavirus-2 infection. Transthoracic echocardiography showed normal morphology and function of the cardiac chambers and excluded valvular and pericardial diseases The recent medical history revealed a hospitalization for the same clinical picture three months earlier with a final diagnosis of non-ST segment elevation myocardial infarction (NSTEMI) with fully normal coronary arteries on a comprehensive diagnostic invasive angiography, including testing for epicardial coronary spasm and microvascular dysfunction. On that occasion, the patient was discharged on dual antiplatelet therapy, which she was still taking at the time of the second hospital admission for chest pain. Of relevance, the patient reported a recent episode of transient edema of the right lower limb and recurrent episodes of dizziness in the last few months.

## Investigations

**Figure 1.**
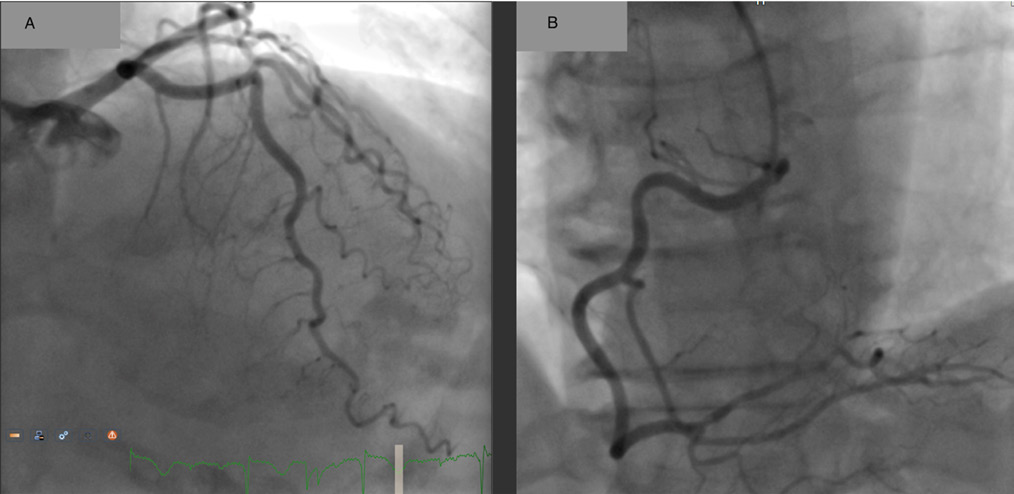
Invasive coronary angiography showing normal vessels: (A) posteroanterior cranial view of left coronary artery and (B) left anterior oblique cranial view of right coronary artery.

**Figure 2.**
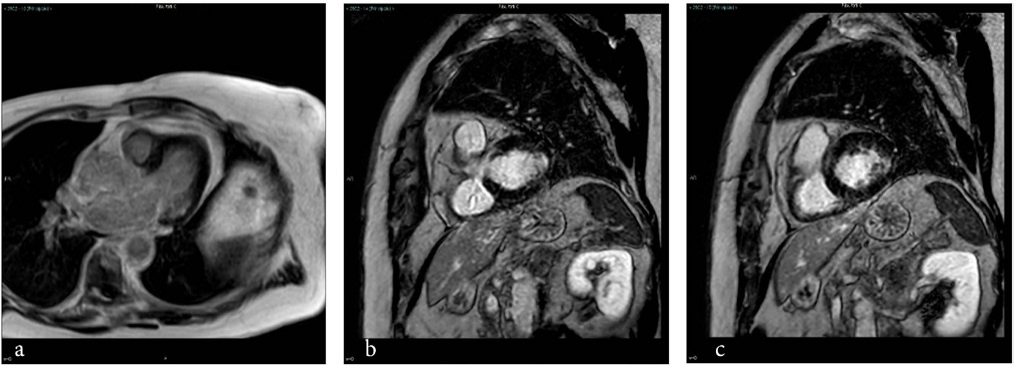
CMR imaging showing subendocardial late gadolinium enhancement in the basal middle lateral segment of the left ventricle: late gadolinium enhancement inversion recovery gradient-echo sequence in three chamber long axis view (a) and short axis view (b), and phase-sensitive inversion recovery sequence in short axis view (c). CMR: cardiac magnetic resonance

**Figure 3.**
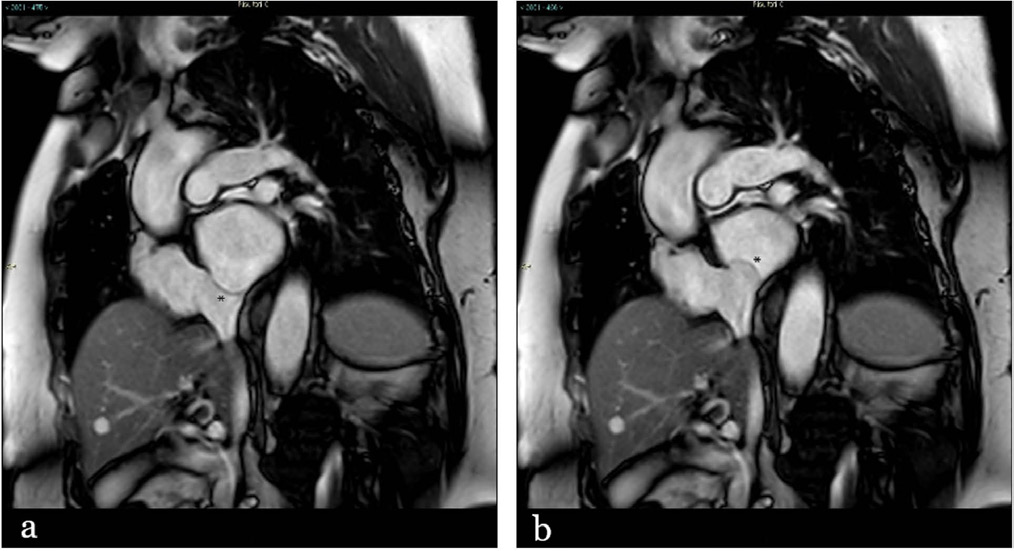
Balanced steady-state free precession sequence CMR imaging in short axis view showing aneurysmal bulging of atrial septal tissue (asterisk) with oscillations into both right (a) and left (b) atrium during the cardiac cycle. CMR: cardiac magnetic resonance

**Figure 4.**
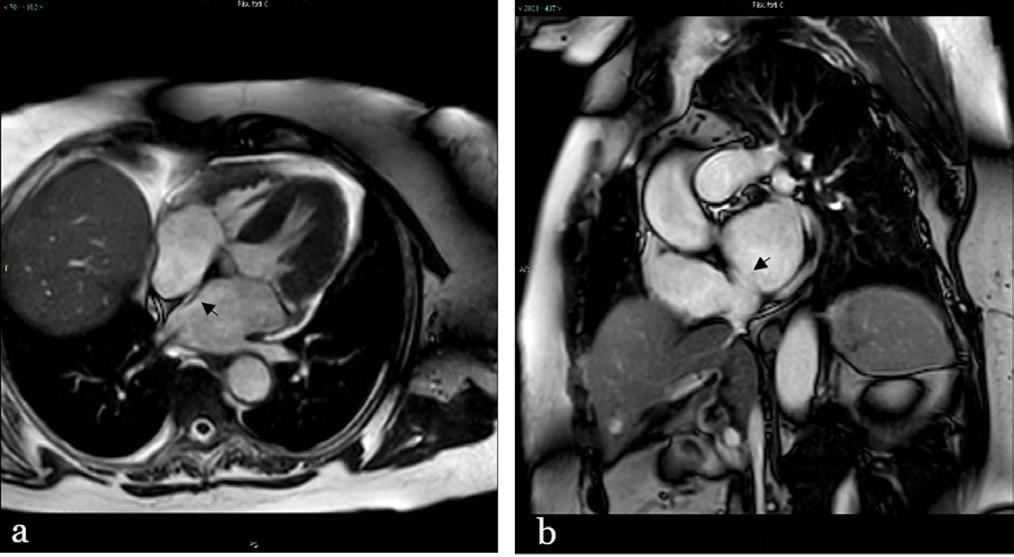
Balanced steady-state free precession sequence CMR in four chamber long axis (a) and short axis (b) view showing a “channel like” pattern of the interatrial septum (arrow, from the left atrium).

**Figure 5.**
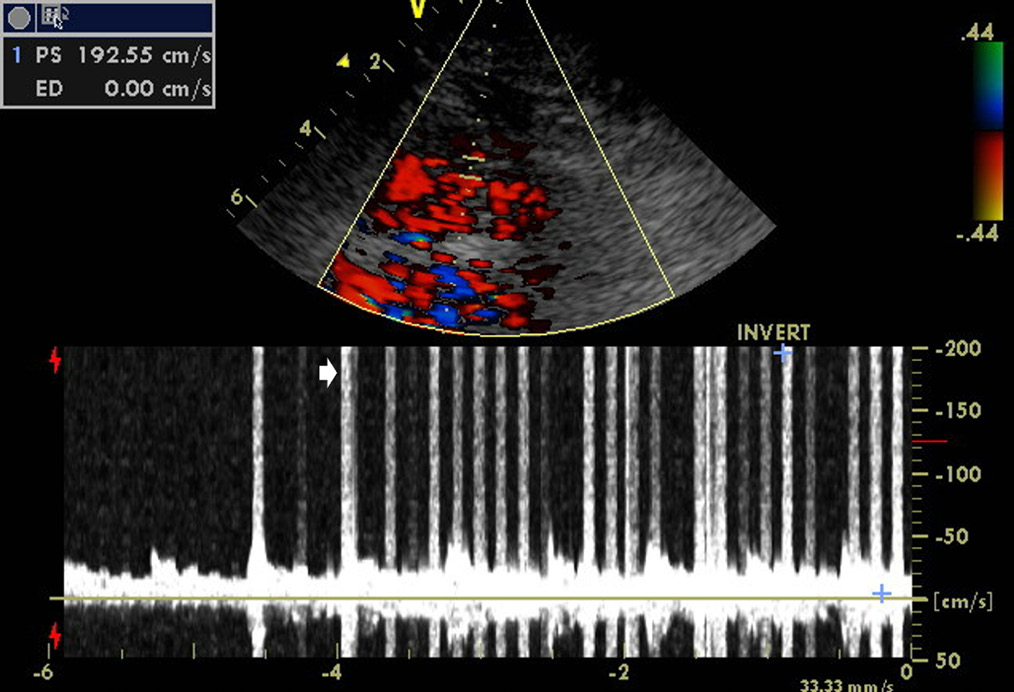
Transcranial Doppler with bubble test showing a large amount of high-intensity transient signals (arrow) representative of microembolic phenomena with “shower” pattern evocated by the Valsalva maneuver at the left middle cerebral artery.

**Figure 6.**
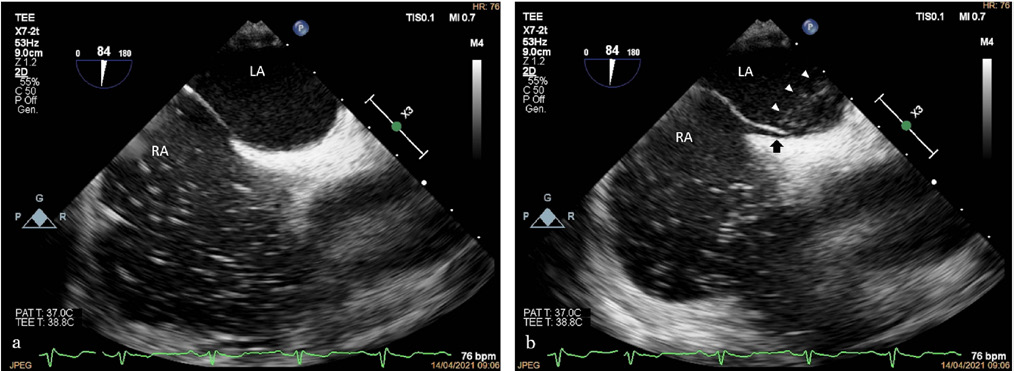
Agitate saline contrast transesophageal study (bubble test) in the bicaval view; (a) while the patient was at rest, only the right atrium was opacified by the bolus of saline bubble contrast; (b) during the Valsalva, maneuver bubbles appear in the left atrium (white arrowheads), crossing the transiently left-side-bulged interatrial septum through a patent foramen ovale (black arrow).

Given the characteristics of the chest pain, we performed an urgent computed tomographic angiography which ruled out both acute aortic syndrome and pulmonary embolism. Therefore, coronary angiography was repeated, confirming normal coronary arteries (Figure 1), and raising the suspicion of myocardial infarction with non-obstructive coronary arteries (MINOCA). To investigate the etiology of myocardial damage, the patient underwent a cardiac magnetic resonance (CMR) imaging that yielded the following findings: myocardial edema in the STIR and T2 mapping sequences, increased T1 mapping values, and subendocardial late gadolinium enhancement (LGE) in the basal-middle lateral segment of the left ventricle, extending up to 50% of the wall thickness (Figure 2). These features were consistent with a recent ischemic pattern. Additionally, CMR showed an atrial septal aneurysm (ASA) (Figure 3) and a channel-like pattern of the interatrial septum (IAS) (Figure 4), raising the suspicion of PFO despite the absence of overt atrial shunt (Qp/Qs ratio = 1). The transesophageal echocardiography (TOE) and transcranial Doppler with bubble test confirmed the presence of a PFO responsible for large right-to-left shunting, with a “shower” pattern of microembolic signals (MES), evocated only by the Valsalva maneuver (Figures 5 and 6 **,** see also Supplementary Video 1); also, TOE excluded other sources of embolic tissue within the life-sided cardiac chambers (*e.g.,* thrombus, valvular material, neoplasm).

Supplementary Video 1.

Then, owing to the anatomic predisposing condition to paradoxical embolism and the recent history of transient oedema of the right calf and episodes, the patient was screened for deep vein thrombosis, which resulted in negative. Moreover, given the large MES detected by TCD and the reported relatively recent history of dizziness, the patient underwent brain magnetic resonance imaging which showed minute multiple areas of impaired signaling of the cerebral white matter, from lacunar ischemic outcomes. We excluded silent paroxysmal atrial fibrillation, as a potential cause of systemic embolism, with ECG telemetry throughout the entire 13 day hospitalization. Finally, although recommended in younger patients, given the history of spontaneous abortions, she was screened for acquired, inherited, and malignancy-associated thrombophilia, which yielded negative results.

## Differential diagnosis

Based on the result of the overall clinical-instrumental evaluation combined with the lack of clinically apparent alternative diagnosis (*e.g.,* myocarditis), MINOCA caused by paradoxical coronary embolism due to a large right-to-left shunting through a PFO was considered the most plausible diagnostic hypothesis.

## Treatment

Following the detection of PFO as explanation for the recurrence of paradoxical coronary embolism despite dual antiplatelet therapy, device closure of PFO was the recommended treatment.

## Outcome and follow-up

The patient was discharged after undergoing interventional percutaneous closure of PFO, with no symptoms or complications at a 3 month follow-up.

## Discussion

According to the Fourth Universal Definition of acute myocardial infarction, the acronym MINOCA (Myocardial Infarction with Non-Obstructive Coronary Arteries) is the current term referring to acute myocardial injury caused by an ischemic mechanism without obstructive coronary atherosclerotic disease (if any stenosis<50%) on invasive angiography.^1^ The most up-to-date registers reported a prevalence of MINOCA by 6–8% among patients diagnosed with acute myocardial infarction, with higher prevalence in the female sex, younger age, and lower cardiovascular risk profile compared to patients with acute myocardial infarction and obstructive coronary arteries.^2^

A broad spectrum of coronary-related pathological mechanisms responsible for MINOCA has been reported. These include plaque disruption/erosion, spontaneous dissection, epicardial spasm, microvascular dysfunction, and coronary embolism. In general, coronary embolism as the mechanism responsible for MINOCA is considered uncommon. However, this may be due to the lack of systematic screening for predisposing conditions such as aortic valve diseases (*e.g.,* fibroelastoma), thrombophilia, silent atrial fibrillation, and right-to-left shunting (*e.g.,* patent foramen ovalis). Paradoxical embolism is a rare case of coronary embolism which may occur in case of right-to-left shunting (*i.e.,* PFO, atrial septal defect, and coronary arteriovenous fistula).^3^ In patient diagnosed with paradoxical coronary embolism, PFO and deep venous thrombosis are the most common causes of intracardiac shunt and embolic source, respectively. However, although the prevalence of PFO in the general population has been estimated as high as 25%, the true incidence of PFO-associated paradoxical coronary embolism is an unknown and likely underestimated phenomenon. Based on scarce pathologic and clinical series and case reports in the literature, a paradoxical embolism through a PFO as the cause of acute myocardial infarction should be suspected and investigated in younger or low cardiovascular risk profile patients, especially with concomitant pulmonary embolism.^4^

A diagnosis of paradoxical coronary embolism is applicable in the case of direct visualization of embolic occlusion on angiography, without embolic source in the left-sided cardiac chambers but, conversely, with evidence of deep vein thrombosis potentially flowing into the systemic circulation through a right-to-left intracardiac shunt. Nevertheless, these criteria are rarely satisfied fully, especially in the case of embolism affecting distal and small coronary vessels, which may be missed on angiography. Since our patient was clinically stable without specific ECG patterns for an acute coronary syndrome, invasive coronary angiography was performed 48–72 h after admission. Consequently, spontaneous coronary recanalization might have occurred during such a time frame, preventing the direct visualization of the embolism. However, Shibata et al have recently proposed a scoring system for diagnosis of definite or probable coronary embolism.^5^ Based on such scoring system, our patient had two minor criteria (*i.e.,* coronary stenosis<25% and the presence of PFO) meaning coronary embolism as probable cause of acute myocardial infarction.

CMR imaging proved highly effective in identifying the underlying pathological mechanism in most MINOCA patients and, therefore, in guiding to choose the most appropriate therapy.^6^ In the recent prospective multicenter Stockholm Myocardial Infarction with Normal Coronaries (SMINC-2) study, 77% of patients with MINOCA received a definitive diagnosis when investigated early with comprehensive CMR imaging.^7^ The updated 2020 European guidelines for management of patients with NSTEMI recommend CMR (Class 1B) in all MINOCA cases with undefined or uncertain cause.^8^

Although TDC or TOE with microbubble test are the gold standard to diagnose PFO [3, 9], a growing body of data in literature underlines the increasing diagnostic value of CMR. CMR imaging provides an accurate detailing of the IAS anatomical variants, presenting as a complete fusion of septum primum and secundum, or vice versa an incomplete fusion determining a “channel-like” tubular morphology, the latter present in 15–38% of the general population and rarely associated with PFO. Moreover, channel length and free limb of the septum primum, are predictive of PFO-associated shunt. The specificity for PFO diagnosis is 100% if three criteria are applied: recognizability of the septum primum flap, continuous interatrial contrast column in correspondence of the tunnel, contrast jet from the column towards the right atrium. Conversely, the diagnostic sensitivity is poor, because of the impossibility of performing the Valsalva maneuver correctly to provoke right-to-left shunting.^9^ In our clinical case, CMR imaging could also detect an ASA, appearing as anomalous mobility of the membrane of the fossa ovalis with excursions greater than 10 mm into both atria during the cardiac cycle. ASA is present in approximately 4% of PFOs and is closely associated with the causal role of a PFO in patients with cryptogenic ischemia, as well as with a worse long-term prognosis in patients treated with antiplatelet against whom treated with the closure of the septal defect with device.^10^ Interestingly, Wöhrle et al showed a not negligible prevalence of subclinical myocardial infarction, as high as 11%, in PFO patients with a first cryptogenic cerebral ischemia who underwent CMR imaging.^11^

## Learning points

- The acronym MINOCA (Myocardial Infarction with Non-Obstructive Coronary Arteries) refers to myocardial infarction with normal or near-normal coronary arteries on invasive angiography. Among patients diagnosed with acute myocardial infarction, 6–8% is labeled as MINOCA.
- The broad spectrum of the pathological mechanisms responsible for myocardial injury in MINOCA makes defining the exact underlying etiology challenging. On the other hand, correctly identifying the culprit mechanism fosters tailored therapies, thus improving the clinical outcome.
- Cardiac magnetic resonance (CMR) imaging proved highly effective in identifying the underlying pathological mechanism in most MINOCAs with uncertain etiology.
